# Diisonitrile Lipopeptides Mediate Resistance to Copper Starvation in Pathogenic Mycobacteria

**DOI:** 10.1128/mbio.02513-22

**Published:** 2022-10-05

**Authors:** John A. Buglino, Yaprak Ozakman, Yao Xu, Farhan Chowdhury, Derek S. Tan, Michael S. Glickman

**Affiliations:** a Immunology Program, Sloan Kettering Institute, Memorial Sloan Kettering Cancer Center, New York, New York, USA; b Chemical Biology Program, Sloan Kettering Institute, Memorial Sloan Kettering Cancer Center, New York, New York, USA; c Tri-Institutional Research Program, Memorial Sloan Kettering Cancer Center, New York, New York, USA; University of Arizona

**Keywords:** *Mycobacterium tuberculosis*, chalkophore, copper, metal resistance, nutritional immunity

## Abstract

Bacterial pathogens and their hosts engage in intense competition for critical nutrients during infection, including metals such as iron, copper, and zinc. Some metals are limited by the host, and some are deployed by the host as antimicrobials. To counter metal limitation, pathogens deploy high-affinity metal acquisition systems, best exemplified by siderophores to acquire iron. Although pathogen strategies to resist the toxic effects of high Cu have been elucidated, the role of Cu starvation and the existence of Cu acquisition systems are less well characterized. In this study, we examined the role of diisonitrile chalkophores of pathogenic mycobacteria, synthesized by the enzymes encoded by the virulence-associated *nrp* gene cluster, in metal acquisition. *nrp* gene cluster expression is strongly induced by starvation or chelation of Cu but not starvation of Zn or excess Cu. Mycobacterium tuberculosis and Mycobacterium marinum strains lacking the *nrp*-encoded nonribosomal peptide sythetase, the *fadD10* adenylate-forming enzyme, or the uncharacterized upstream gene *ppe1* are all sensitized to Cu, but not Zn, starvation. This low Cu sensitivity is rescued by genetic complementation or by provision of a synthetic diisonitrile chalkophore. These data demonstrate that diisonitrile lipopeptides in mycobacteria are chalkophores that facilitate survival under Cu-limiting conditions and suggest that Cu starvation is a relevant stress for M. tuberculosis in the host.

## INTRODUCTION

Bacterial pathogens express complex virulence programs to ensure their survival within the host, including factors that confer resistance to or modify host immunity. Metal acquisition and resistance are one set of mechanisms used by phylogenetically diverse bacterial pathogens (1). Intracellular pathogens live in a nutrient- and metal-limited niche and require active acquisition of metals critical for enzymatic function. For example, host cells impose iron limitation on pathogens, a host strategy that is counteracted by microbial siderophores, high-affinity iron-binding molecules critical for virulence across pathogens (2). Mycobacterium tuberculosis lacking the iron siderophore mycobactin due to either genetic ablation of mycobactin biosynthesis (3), interruption of export (4), or chemical inhibition (5) is attenuated in infection models, as is the case for multiple other bacterial pathogens (6).

Despite the extensive characterization of iron acquisition systems, the relevance of acquisition of other trace metals is less well defined. Zinc and copper are essential nutrients and serve as a enzymatic cofactors, but scavenging systems for these metals are less well characterized (7). There is emerging evidence that pathogens use Zn acquisition systems during infection, through either-the elaboration of Zn-scavenging molecules, transport systems for Zn (8), or import of host-derived Zn containing molecules (9–11). It was recently shown that yersiniabactin, previously characterized as an iron siderophore, also binds Cu (12, 13) and Zn (14) and that the yersiniabactin system is active during infections (12, 14–17).

In addition to metal limitation, the host also deploys copper and zinc as antimicrobial effectors to limit pathogen growth (18). Macrophages deposit Cu and Zn into the phagosome, and there is substantial evidence that M. tuberculosis experiences high Cu and Zn stress during infection (18–22). Recently, we identified a new pathway in M. tuberculosis that jointly controls nitric oxide and Cu resistance governed by the Rip1 intramembrane protease and the cytoplasmic PdtaS/PdtaR two-component system (19). This study identified the *nrp* gene cluster (*rv0097* to *rv0101*) as a target of Rip1/PdtaS/R regulation and demonstrated that expression of the *nrp* locus was the critical determinant of NO resistance. However, despite the important role of Rip1/PdtaS/R in controlling resistance to Cu toxicity, the *nrp* cluster was not important for resistance to high Cu (19).

**FIG 1:**
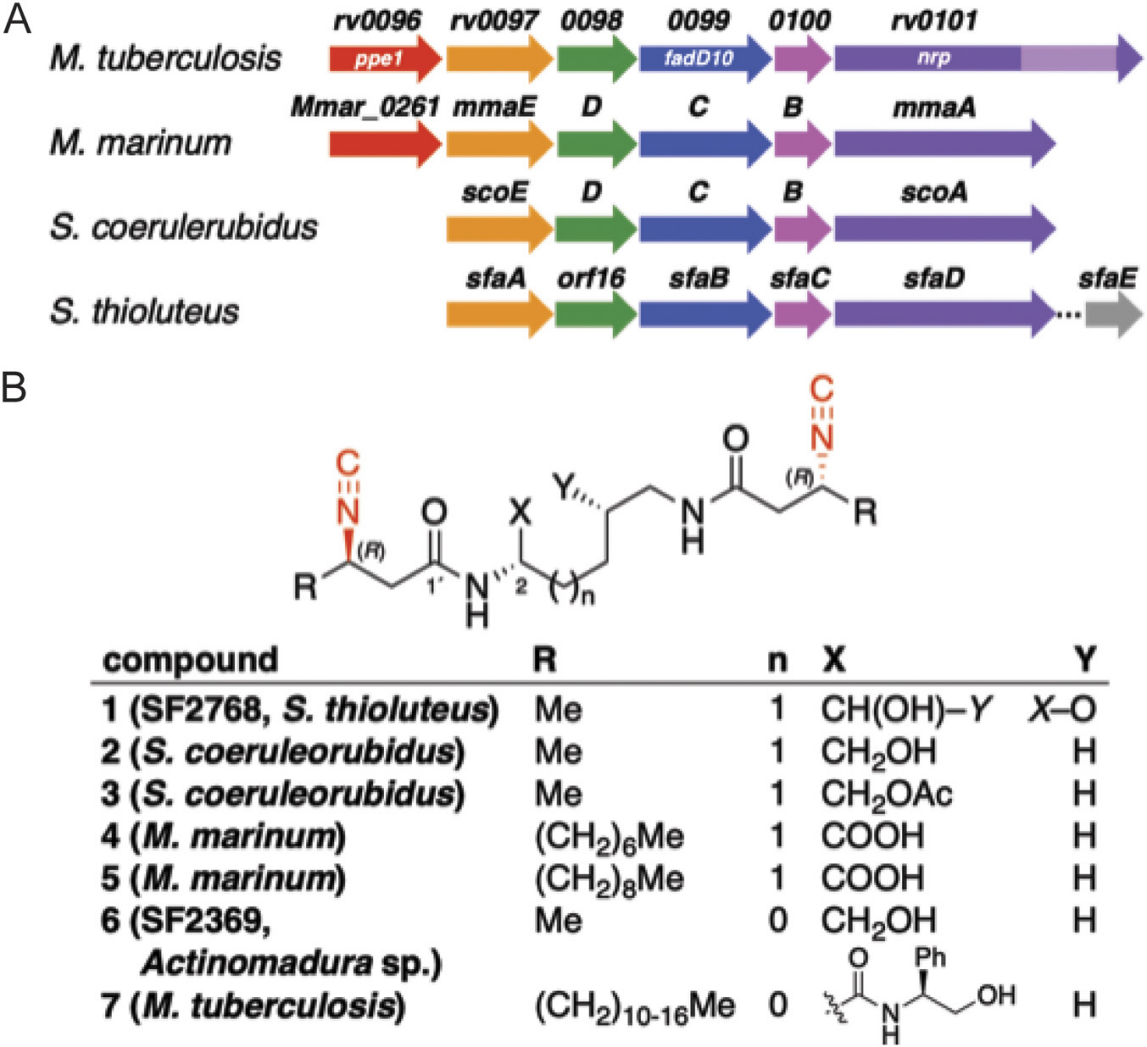
Biosynthetic gene clusters and structures of diisonitrile lipopeptide natural products. (A) Biosynthetic gene clusters that produce diisonitrile lipopeptide natural products. The M. tuberculosis NRPS encoded by *nrp* (*rv0101*) contains an additional module (light purple) that is proposed to install a C-terminal phenylalanine residue in the corresponding diisonitrile 7. The mycobacterial gene clusters include an upstream predicted transmembrane protein, PPE1 (red). The *S. thioluteus* gene cluster contains an additional downstream tailoring enzyme, SfaE (gray), which is proposed to hydroxylate the lysine backbone in SF2768 (compound 1), leading to ring formation. (B) Structures of diisonitrile lipopeptides produced as natural products (compounds 1, 6, 7) or by heterologous expression of diisonitrile biosynthetic enzymes in E. coli (compounds 2 to 5). Isonitrile motifs (red) engage in copper chelation.

The *nrp* gene cluster (*rv0097* to *rv0101*), named for the nonribosomal peptide synthetase (NRPS) encoded by *rv0101*, has been implicated in M. tuberculosis virulence in multiple studies (19, 23, 24). M. tuberculosis lacking *nrp* is attenuated for growth in the lung during early infection but has no persistent virulence defect at later time points. Although these studies implicate the biosynthetic product of the *nrp* gene cluster in pathogenesis, the identity of this natural product was unknown. Recent studies using heterologous expression of the orthologous *nrp* gene clusters from Streptomyces thioluteus, Streptomyces coeruleorubidus, and Mycobacterium marinum (without *Mmar_0261*/*ppe1*) revealed a series of diisonitriles (compounds 1 to 5) that differed in structure based on the origin of the NRPS (Fig. 1A, B) (25, 26). Examination of the metal binding specificity of the diisonitrile products from heterologous expression (26) or synthesis (27) revealed tight binding to Cu. Further, products of the *S. thioluteus nrp* cluster expressed in Streptomyces lividans can directly mediate Cu uptake, indicating that diisonitriles function as chalkophores (26). Most recently, the structure of the corresponding M. tuberculosis NRPS diisonitrile product (compound 7 in Fig. 1B) was identified, and it has several structural differences from the *Streptomyces* and M. marinum diisonitriles. Whereas diisonitriles 1 to 5 have lysine-based cores, M. tuberculosis diisonitrile 7 has a shorter ornithine-based core, which is also found in the natural product SF2369 (compound 6) (28), a C-terminal phenylalanine extension, attributed to an additional NRPS module found only in Rv0101, and very long (C_14_ to C_20_) β-isocyano acyl chains (Fig. 1) (24). However, the metal binding specificity of this M. tuberculosis diisonitrile is not known.

The physiologic roles of the diisonitrile products of the *nrp* locus in mycobacteria, in particular their roles in metal acquisition and detoxification, are unknown. In mycobacteria, the *nrp* cluster includes the *ppe1* gene (*rv0096*) as a conserved feature, but the functional importance of *ppe1* is unknown. In this study, we used genetic deletion of the *nrp* gene and the upstream genes *ppe1* and *fadD10*, coupled with assays of metal starvation, to deduce the primary function of the *nrp* cluster. We show that these elements of the *nrp* gene clusters are required for resistance to starvation of Cu but not other metals, establishing the products of these gene clusters as chalkophores of pathogenic mycobacteria. Further, we show that a synthetic diisonitrile can rescue growth of *nrp* gene cluster mutants under Cu restriction, indicating that mycobacteria can accept this nonnative diisonitrile as a chalkophore.

## RESULTS

### The *nrp* gene cluster is transcriptionally induced under copper starvation or chelation but does not respond to Zn starvation.

**FIG 2:**
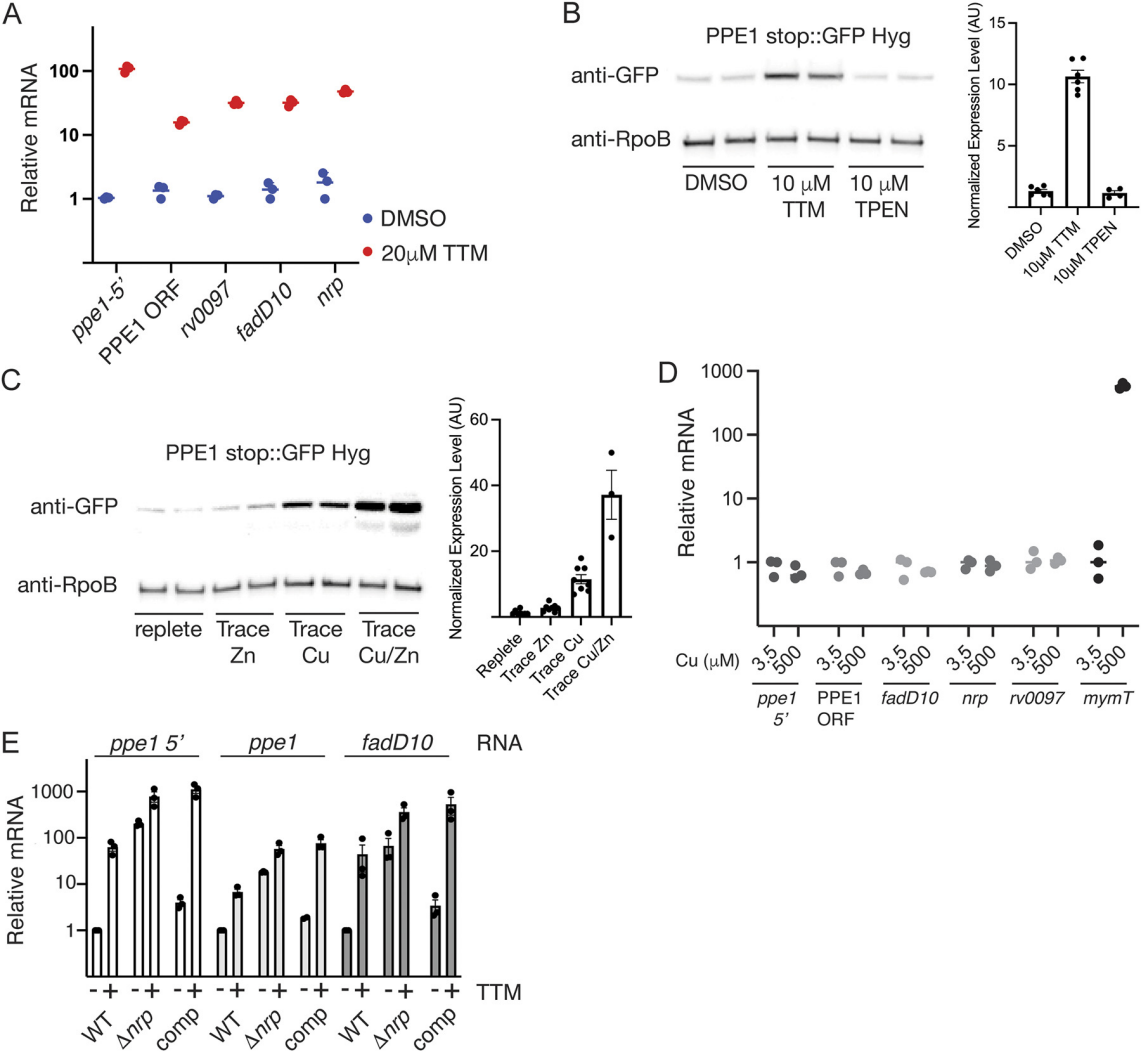
The chalkophore gene cluster is induced under low-copper conditions. (A) Normalized mRNAs from the chalkophore cluster genes, indicated on the *x* axis, measured by RT-qPCR in M. tuberculosis treated with DMSO (vehicle [blue]) or 20 μM tetrathiomolybdate (TTM). Each point represents a biologic replicate, and values are normalized to *sigA* under the DMSO condition. (B) Immunoblotting with anti-GFP or anti-RpoB antibodies of protein lysates from a strain with a functional C-terminal translational fusion of GFP to the C terminus of PPE1 (PPE1 stop::GFP Hyg), treated with DMSO, 10 μM TTM, or 10 μM TPEN. Quantitation of replicate immunoblots is given in the bar graph. GFP signal was normalized to RpoB in the same lane and then relative to DMSO, which is set at 1. AU, arbitrary units. (C) Immunoblotting of biologic duplicates as in panel B in cells grown in metal-replete medium or medium lacking Zn, Cu, or Cu/Zn and corresponding quantitation. GFP signal was normalized to RpoB in the same lane and then relative to replete medium, which is set at 1. (D) mRNAs of the chalkophore cluster are not induced by high Cu. The indicated mRNAs were quantitated as in panel A in wild-type M. tuberculosis grown in 3.5 or 500 μM Cu. Each point is the average of technical triplicates for 3 biologic replicates. (E) Chalkophore transcription is autoregulated. Shown are results for mRNA quantitation by RT-qPCR of *ppe1-5′*, *ppe1*, or *fadD10* in wild-type M. tuberculosis (WT), M. tuberculosis Δ*nrp*, or M. tuberculosis Δ*nrp* plus *nrp* (comp) with or without treatment with TTM. Error bars indicate SEM.

Our prior experiments determined that the *nrp* gene cluster is induced by nitric oxide and is a target of the Rip1/PdtaS/R regulatory cascade that controls NO resistance (19). However, although this Rip1/PdtaS/PdtaR system controls Cu resistance, the *nrp* gene cluster was not involved in the Cu resistance arm of the pathway (19), despite the high Cu affinity of related diisonitrile products produced by *Streptomyces* (26, 27), suggesting an alternative role in Cu homeostasis. To understand the function of the *nrp* gene cluster in the mycobacterial metal response, we measured the levels of the mRNAs encoding multiple proteins in the cluster from *rv0096* through *rv0101* (NRPS) under conditions of metal limitation. We first treated M. tuberculosis with tetrathiomolybdate (TTM), a Cu chelator, and measured each mRNA by real-time quantitative PCR (RT-qPCR). All genes in the cluster were induced by TTM 10- to 100-fold compared to untreated cells (Fig. 2A). The *ppe1-5′* RNA at the 5′ end of the PPE1 open reading frame (ORF), previously identified as induced by nitric oxide (19), was the most highly induced by TTM (Fig. 2A). To measure the PPE1 protein, we engineered an M. tuberculosis strain with a functional (Fig. S5B) C-terminal green fluorescent protein (GFP) fusion to the chromosomal copy of PPE1 and treated it with TTM. TTM treatment strongly induced PPE1 protein expression (12-fold) compared to vehicle-treated cells (Fig. 2B). Treatment with the Zn selective chelator *N*,*N*,*N′*,*N′*-tetrakis(2-pyridylmethyl)ethylenediamine (TPEN) at a concentration known to impose Zn limitation on M. tuberculosis (29) did not alter PPE1-GFP expression, suggesting that the effect is specific to Cu chelation (Fig. 2B). To confirm that the effect of TTM was due to Cu limitation, we depleted mycobacterial 7H9 medium of all metals using Chelex ion exchange resin and then reconstituted it with defined metal concentrations, leaving out either Cu or Zn. We confirmed that our Zn-depleted medium imposed Zn limitation by measuring the induction of *ctpV*, previously reported to be one of the most strongly induced genes in M. tuberculosis exposed to low Zn (29). Low Cu weakly induced *ctpV*, but, consistent with prior reports, low Zn strongly induced *ctpV* RNA, indicating that our zinc-depleted medium imposed zinc starvation (see Fig. S1 in the supplemental material). When bacteria were grown in Cu-depleted medium, PPE1 protein was strongly induced, but Zn starvation alone had no effect (Fig. 2C). We observed that starvation of Zn did further induce PPE1 when Cu was limiting, suggesting that Zn influences expression of the *nrp* locus only when Cu is limiting (Fig. 2C).

10.1128/mbio.02513-22.1 FIG S1 Low-zinc conditions include *ctpV*. Shown are normalized *ctpV* mRNA values as reported previously (J. A. Buglino, G. D. Sankhe, N. Lazar, J. M. Bean, et al., Elife 10:e65351, 2021, https://doi.org/10.7554/eLife.65351) in M. tuberculosis grown in metal-replete medium or medium lacking Cu or Zn. RNA was harvested after 2 days of growth in the indicated media. Values for replete media are set to 1, and each point represents a biologic replicate. *, *P* < 0.05; ***, *P* < 0.0001. Error bars represent SD. Download FIG S1, TIF file, 0.1 MB. Copyright © 2022 Buglino et al. 2022 Buglino et al. https://creativecommons.org/licenses/by/4.0/ This content is distributed under the terms of the Creative Commons Attribution 4.0 International license.

10.1128/mbio.02513-22.5 FIG S5 (A) Serial dilutions (10-fold) of M. tuberculosis WT, Δ*ppe1*, and complemented strains on agar medium with no addition, 1 mM BCS, or 0.04 mM TTM. (B) Serial dilutions (10-fold) of WT M. tuberculosis or M. tuberculosis with a C-terminal GFP fusion to the chromosomal PPE1 protein, grown on agar medium with no addition, 1 mM BCS, or 0.04 mM TTM. Download FIG S5, TIF file, 2.4 MB. Copyright © 2022 Buglino et al. 2022 Buglino et al. https://creativecommons.org/licenses/by/4.0/ This content is distributed under the terms of the Creative Commons Attribution 4.0 International license.

The M. tuberculosis regulon induced by high Cu has been extensively studied (20, 21, 30), and the *nrp* gene cluster has not been identified as responsive to high Cu. To confirm these prior results, we treated cells with 500 μM CuSO_4_ and measured the mRNA of each gene in the *nrp* cluster. Consistent with prior data, high Cu did not induce any gene in the *nrp* cluster, whereas *mymT*, previously identified as regulated by the Cu-responsive repressor RicR (20, 31), was highly induced (Fig. 2D). These data indicate that *nrp* gene cluster expression is primarily responsive to Cu but not Zn limitation, imposed by either chelation or metal starvation, but not Cu excess.

### Chalkophore biosynthesis is transcriptionally autoregulated.

Many bacterial regulons are subject to feedback regulation such that the biosynthetic end product, or an intermediate, represses expression of the genes that lead to synthesis of that product. To determine whether expression of the chalkophore cluster is affected by the production of the diisonitrile product, we measured the RNAs of *ppe1-5′*, *ppe1*, and *fadD10* in an M. tuberculosis strain lacking the *nrp* gene (Fig. S2), which should not produce chalkophores, with and without TTM. In wild-type cells, TTM induced expression of all three mRNAs, as reported above (Fig. 2E). In the Δ*nrp* strain, all three mRNAs were induced without Cu chelation to a level similar to that in wild-type cells with chelation (Fig. 2E). Treatment with TTM had a small incremental inductive effect on each RNA, and complementation with the wild-type *nrp* gene restored the inducibility pattern of wild-type cells, indicating that these phenotypes are due to loss of *nrp* and chalkophore biosynthesis. These data strongly indicate that the expression of chalkophore biosynthesis machinery is repressed by the diisonitrile chalkophore product, either directly or indirectly.

10.1128/mbio.02513-22.2 FIG S2 Confirmation of M. tuberculosis Erdman Δ*nrp*, Δ*ppe1*, and Δ*fadD10* genotypes. Each gel is a PCR amplification of the wild type or the indicated mutant using primers flanking the gene of interest. See Materials and Methods for details. Download FIG S2, TIF file, 2.8 MB. Copyright © 2022 Buglino et al. 2022 Buglino et al. https://creativecommons.org/licenses/by/4.0/ This content is distributed under the terms of the Creative Commons Attribution 4.0 International license.

### Loss of *nrp* confers sensitivity to copper chelation or starvation but not zinc chelation or starvation.

**FIG 3:**
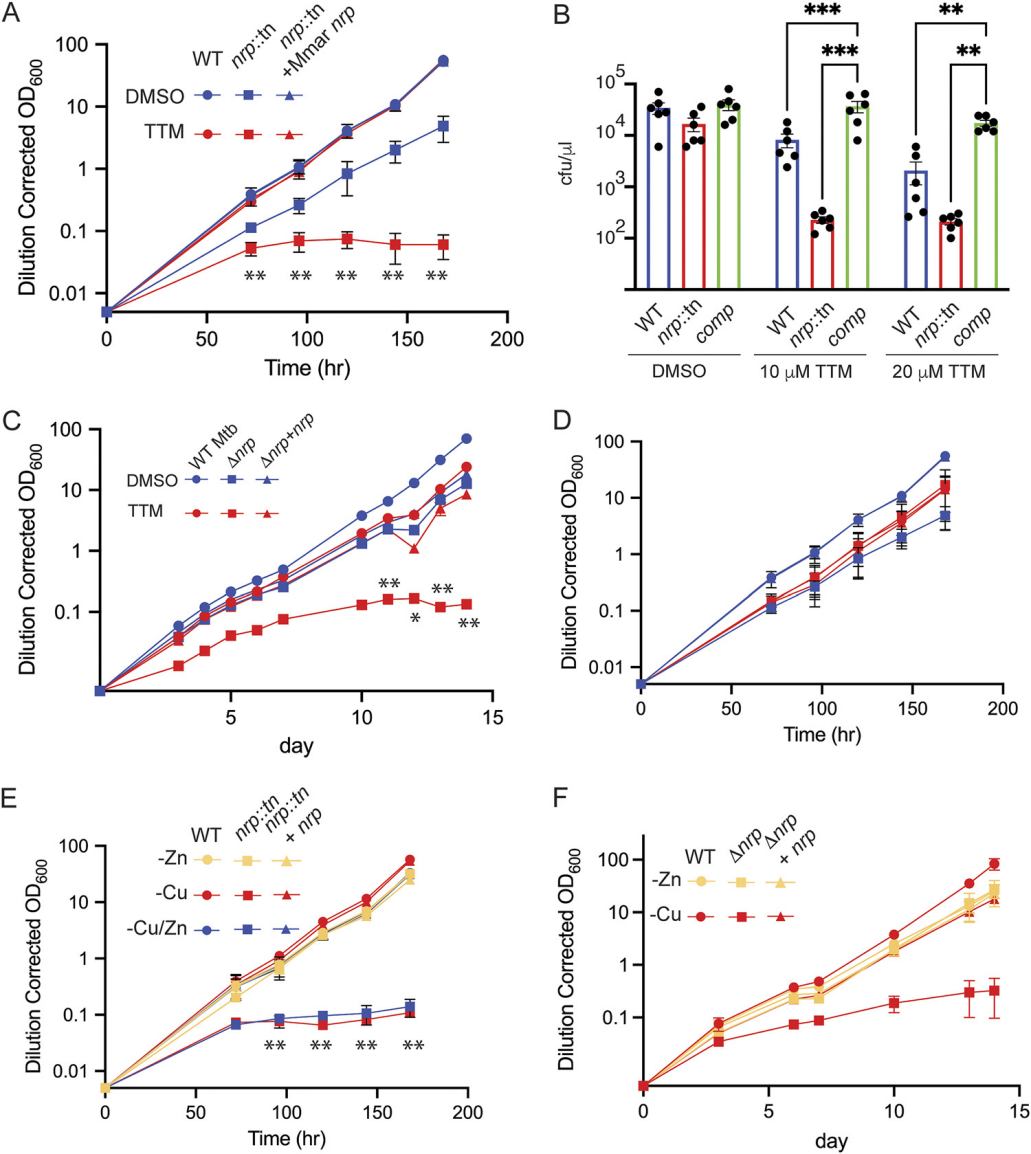
Loss of *nrp* confers sensitivity to copper chelation or limitation. (A)Wild-type M. marinum (circles), M. marinum *nrp*::*tn* (squares), M. marinum *nrp*::*tn* plus M. marinum *nrp* (triangles) treated with vehicle DMSO (blue) or 10 μM TTM (red). Dilution-corrected OD of a continuous growth curve is shown, with each point the mean of triplicate cultures and error bars displaying SEM. **, *P* < 0.01 by unpaired test for each time point of *nrp*::tn versus wild-type or complemented strain. (B) Bacterial killing assays of the same strains as in panel A, treated with 10 or 20 μM TTM as described in Materials and Methods. Bacterial survival was quantitated on agar medium without TTM. Each dot represents a biologic replicate. ***, *P* < 0.001; **, *P* < 0.01. (C) Extended growth curves of the indicated M. tuberculosis strains with (red) or without (blue) TTM. **, *P* < 0.01; *, *P* < 0.02. (D) Growth of the same M. marinum strains as in panel A with 10 μM Zn chelator TPEN. (E) Growth of M. marinum strains in medium lacking Cu and Zn (blue), Cu alone (red), or Zn alone (yellow). **, *P* < 0.0001. (F) Growth of M. tuberculosis strains in medium lacking Cu (red) or Zn alone (yellow). Error bars for all panels indicate SD and when not visible are within the symbol.

To determine the contribution of isonitrile lipopeptide biosynthesis to mycobacterial growth under metal-limiting conditions, we tested M. marinum (*nrp*::*tn*) or M. tuberculosis (Δ*nrp*) lacking *nrp* for sensitivity to TTM. Wild-type M. marinum growth was unaffected by 20 μM TTM, whereas growth of M. marinum lacking *nrp* arrested after several doublings (Fig. 3A). Complementation of M. marinum *nrp*::*tn* with M. marinum *nrp* restored TTM resistance to wild-type levels.

Bathocuproinedisulfonic acid (BCS) is a copper chelator with an association constant for Cu(I) of 10^19.8^ M^−2^ (32) in 2:1 stoichiometry. Addition of BCS did not affect growth of wild-type M. marinum but arrested growth of M. marinum lacking *nrp* (Fig. S3). This is consistent with a previous study that showed that a synthetic diisonitrile chalkophore, related to the predicted structure of the M. marinum natural product diisonitrile, has much higher affinity for Cu(I) than does BCS (27). To confirm that the effect of BCS is mediated by Cu starvation, we supplemented BCS with Cu, Zn, Mn, and Fe and observed that only Cu could reverse the growth limitation of *nrp*-deficient M. marinum by BCS (Fig. S3). In addition to limiting growth, TTM treatment was lethal for *nrp*-deficient M. marinum, a phenotype that was also rescued by genetic complementation (Fig. 3B).

10.1128/mbio.02513-22.3 FIG S3 The WT (circles) and M. marinum *nrp*::*tn* (squares) were grown in the copper chelator BCS either without added metal or with an equimolar concentration of Mn, Zn, Cu, or Fe as indicated in the legend. The *y* axis represents a dilution-corrected OD_600_ value to maintain logarithmic growth as described in Materials and Methods. Download FIG S3, TIF file, 0.8 MB. Copyright © 2022 Buglino et al. 2022 Buglino et al. https://creativecommons.org/licenses/by/4.0/ This content is distributed under the terms of the Creative Commons Attribution 4.0 International license.

For M. tuberculosis, growth of the wild-type Erdman strain was also unaffected by TTM, but M. tuberculosis Δ*nrp* ceased replication after several doublings, a phenotype that was complemented by reintroduction of *nrp* (Fig. 3C). We confirmed that M. tuberculosis lacking *nrp* was sensitized to both BCS and TTM on agar media (Fig. S4). To test whether chelation of zinc had a similar effect, we repeated this experiment with the Zn chelator TPEN. Addition of 10 μM TPEN had no effect on growth of either wild-type M. marinum or M. marinum *nrp*::*tn*, indicating that the M. marinum NRPS (*mmaA*) function is not required to resist Zn chelation (Fig. 3D).

10.1128/mbio.02513-22.4 FIG S4 M. tuberculosis lacking *nrp* is sensitive to BCS and TTM. Each agar plate has triplicate spottings of 10-fold dilutions of the indicated M. tuberculosis strains on agar containing no addition, BCS, or TTM at the indicated concentrations. Download FIG S4, TIF file, 2.6 MB. Copyright © 2022 Buglino et al. 2022 Buglino et al. https://creativecommons.org/licenses/by/4.0/ This content is distributed under the terms of the Creative Commons Attribution 4.0 International license.

To extend these results, we tested whether isonitrile lipopeptide biosynthesis is required for growth under Cu-limiting conditions using the media preparation strategy described above. Although wild-type cells grew in media depleted of both copper and zinc, both M. marinum (Fig. 3E) and M. tuberculosis lacking *nrp* (Fig. 3F) were unable to grow in medium without added Cu and Zn, a phenotype that was complemented by wild-type *nrp* in both species. Supplementation of this medium with Zn did not restore growth of *nrp*-deficient cells but supplementation with Cu did, indicating that *nrp* is required for growth in low Cu but not low Zn (Fig. 3E and F), recapitulating the results with Cu and Zn chelation. These experiments suggest that *nrp* function is uniquely required for bacterial growth under Cu-limiting conditions and that mycobacterial chalkophores function in Cu but not Zn acquisition.

### Loss of FadD10 confers sensitivity to low copper.

**FIG 4:**
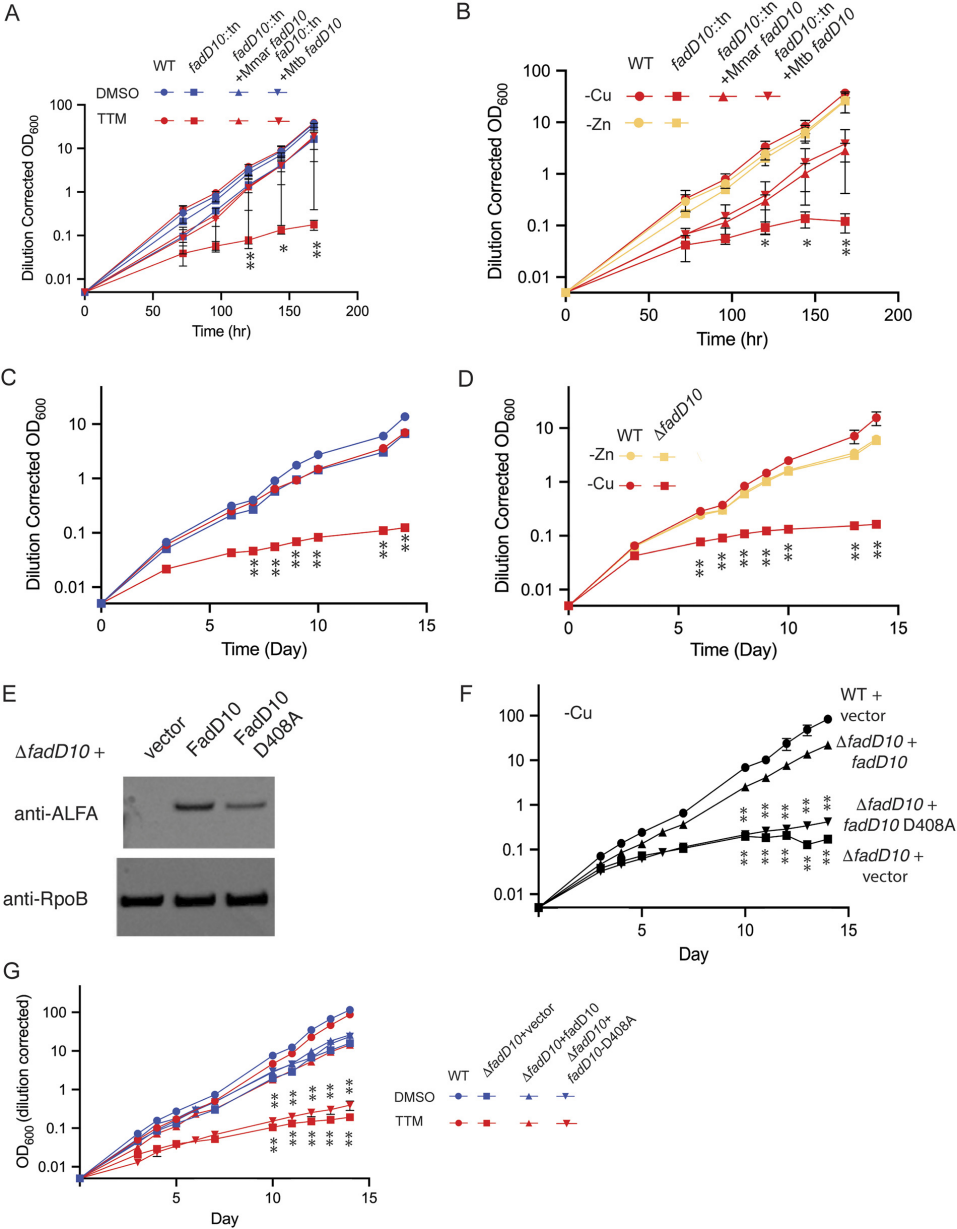
FadD10 activity is required for low copper tolerance. (A) Wild-type M. marinum (circles), M. marinum *fadD10*::*tn* (squares), M. marinum *fadD10*::*tn* plus M. marinum *fadD10* (triangles), and M. marinum *fadD10*::*tn* plus M. tuberculosis *fadD10* (inverted triangles) treated with the vehicle DMSO (blue) or 10 μM TTM (red). Dilution-corrected OD of a continuous growth curve (see Materials and Methods) is shown, with each point the mean of triplicate cultures and error bars displaying SEM. **, *P* < 0.01 by unpaired test for each time point of *fadD10*::*tn* versus the wild type. (B) FadD10 is required for growth in low Cu but not low Zn. The indicated strains were grown in medium lacking either Cu (red) or Zn (yellow). **, *P* < 0.01 by unpaired test for each time point of *fadD10*::*tn* versus the wild type. Error bars display SEM. (C) M. tuberculosis wild type (circles) or M. tuberculosis Δ*fadD10* (squares) treated with either DMSO (blue) or TTM (red). **, *P* < 0.01 by unpaired test for each time point of Δ*fadD10* versus the wild type. Error bars display SEM. (D) M. tuberculosis wild type (circles) or M. tuberculosis Δ*fadD10* (squares) grown in medium lacking Zn (yellow) or Cu (red). **, *P* < 0.01 by unpaired test for each time point of Δ*fadD10* versus the wild type. Error bars display SD. (E) Whole-cell protein lysates of M. tuberculosis Δ*fadD10* with either plasmid vector or plasmids encoding C-terminally ALFA-tagged FadD10 or FadD10-D408A were separated by SDS-PAGE and probed with antibodies to RpoB as a loading control or an anti-ALFA tag nanobody. (F) Low-Cu growth curves of the indicated strains from panel E. **, *P* < 0.01 by unpaired test for each time point of *fadD10*::tn+vect versus *fadD10*::tn+*fadD10* or *fadD10*::tn+*fadD10* versus *fadD10*::tn+*fadD10*-D408A. Error bars display SEM. (G) Growth of the indicated strains as in panel F with or without TTM. Significance comparisons are the same as in panel F. Error bars display SEM.

Although the *nrp* gene cluster can direct isonitrile chalkophore biosynthesis in heterologous bacteria (25, 26), the individual roles of the genes in the chalkophore cluster for diisonitrile biosynthesis are not known. FadD10 (encoded by *rv0099*) is an upstream adenylate-forming enzyme (33) that is involved in synthesis and coupling of the characteristic β-isocyano fatty acid chains in the natural products (25, 34). To determine whether FadD10 (35) is required for resistance to copper starvation, we tested M. marinum and M. tuberculosis mutants lacking *fadD10*. M. marinum *fadD10*::*tn* was severely growth restricted in the presence of 10 μM TTM, a phenotype that was complemented by the wild-type M. marinum or M. tuberculosis *fadD10* gene (Fig. 4A). M. marinum *fadD10*::*tn* also could not grow in Cu-depleted medium, a phenotype that was also genetically complemented by M. marinum or M. tuberculosis *fadD10*, whereas loss of *fadD10* had no effect on growth without Zn (Fig. 4B). We observed a similar effect with M. tuberculosis, in which a Δ*fadD10* mutant was deficient for growth in TTM or low Cu but not low Zn (Fig. 4C and D). To determine whether the activity of FadD10 is required for low-Cu growth, we mutated aspartate 408 to alanine (D408A), as this residue directly interacts with the ribose 3′-hydroxyl of the cognate fatty acyl-AMP reaction intermediate in the FadD10 structure (35). To confirm protein expression, we inserted an ALFA tag (36) at the C termini of both wild-type FadD10 and FadD10(D408A). The two proteins were expressed to similar levels as detected by an ALFA-specific nanobody (Fig. 3E). Wild-type FadD10 ALFA fully rescued M. tuberculosis Δ*fadD10* under conditions of low Cu growth and TTM chelation (Fig. 4F and G). However, FadD10(D408A) could not rescue growth under either condition, suggesting that FadD10 substrate binding is required for chalkophore biosynthesis.

### Chemical rescue of *nrp* deficiency with synthetic diisonitrile.

The data above indicate that loss of two genes in the *nrp* operon, *nrp* (*rv0101*) itself and the upstream *fadD10* (*rv0099*), encoding an adenylate-forming enzyme, confers sensitivity to low copper or Cu chelation. These phenotypes are genetically complemented and are therefore due to deficiency in NRPS or FadD10 function. Prior work demonstrated that the *nrp* gene cluster from *Streptomyces* or M. marinum, when expressed in Escherichia coli, directs synthesis of a diisonitrile lipopeptides that bind Cu with high affinity (26). Total synthesis of the Streptomyces thioluteus-derived diisonitrile SF2768 and related linear congeners confirmed that these molecules bind Cu(I) in a 2:1 stoichiometry with a Cu affinity that exceeds that of BCS, but they failed to demonstrate strong Zn binding (27). To test whether the low-Cu phenotype of mycobacterial cells lacking *nrp* or *fadD10* is due to deficiency of the diisonitrile product, we attempted to rescue the low-Cu phenotype in *trans* with a synthetic chalkophore.

**FIG 5:**
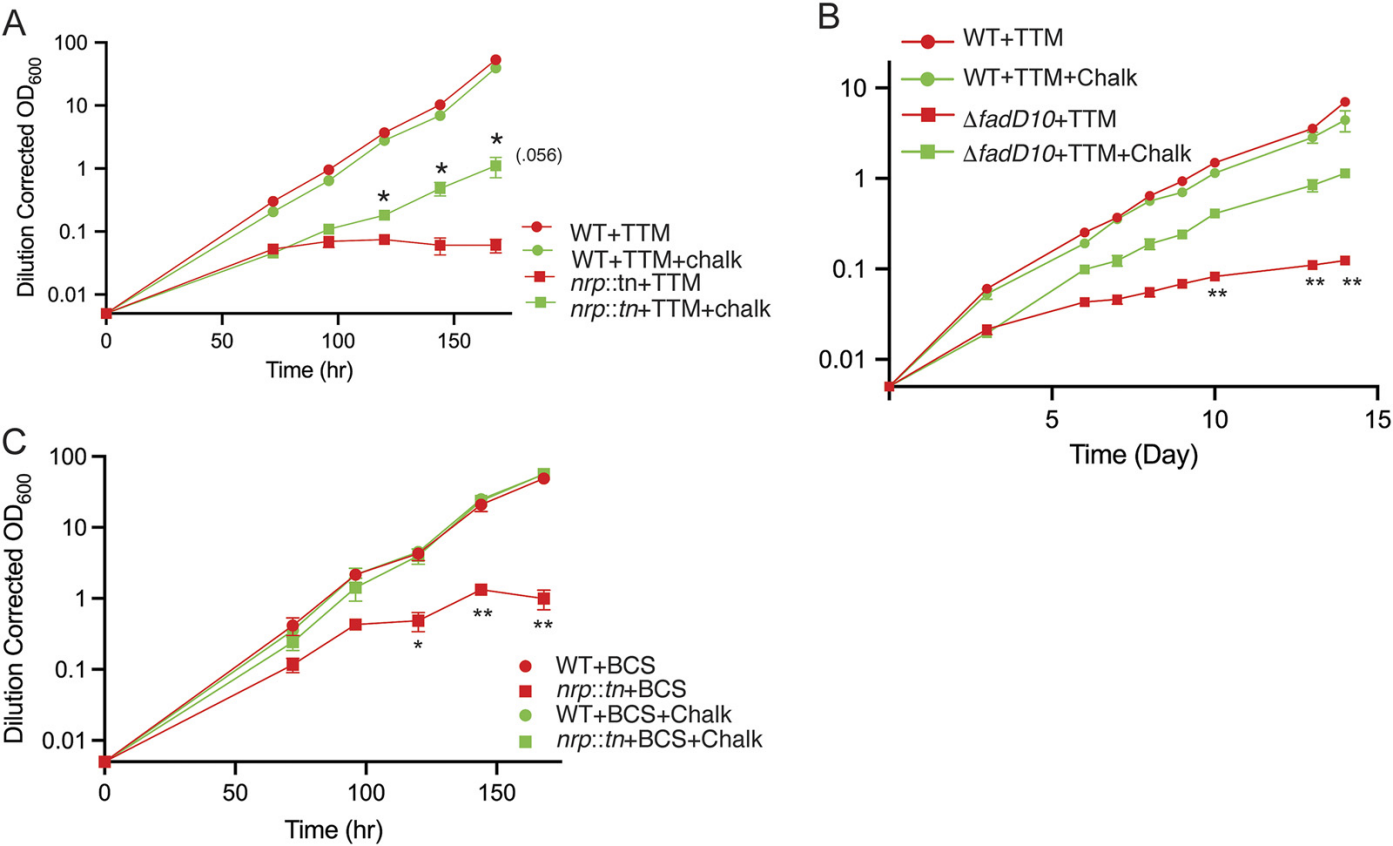
Synthetic chalkophore rescues genetic *fadD10* or *nrp* sensitivity to copper chelation. A. Growth of wild-type M. marinum (circles) or M. marinum *nrp*::*tn* (squares) with TTM either with (green) or without (red) 10 μM synthetic compound 3. (B) Growth of wild-type M. tuberculosis (circles) or M. tuberculosis Δ*fadD10* (squares) with TTM either with (green) or without (red) 10 μM synthetic compound 3. (C) Growth of wild-type M. marinum (circles) or M. marinum *nrp*::*tn* (squares) with BCS either with (green) or without (red) 10 μM synthetic compound 3. For all panels, statistical comparisons were done by unpaired *t* test for mutant strain (i.e., *nrp* or *fadD10*) with or without added chalkophore. *, *P* < 0.05; **, *P* < 0.01. Error bars indicate SEM.

Because mycobacterial chalkophore products have not been synthesized, we used a lysine-derived congener (compound 4 in reference 27 and compound 3 in Fig. 1B) with the caveat that this molecule may not be used efficiently by M. marinum or M. tuberculosis, particularly given that it has 4-carbon β-isonitrile side chains, much shorter than those attributed to the mycobacterial diisonitriles (24, 25). Despite this caveat, addition of 10 μM synthetic diisonitrile partially rescued TTM-imposed growth restriction of M. marinum lacking *nrp* (Fig. 5A). The TTM-imposed growth restriction of M. tuberculosis lacking *fadD10* was also substantially reversed by the synthetic chalkophore (Fig. 5B). The synthetic chalkophore also fully rescued M. marinum lacking *nrp* from growth restriction by BCS (Fig. 5C). These results demonstrate that the sensitivity of mycobacteria lacking *fadD10* or *nrp* to Cu starvation is due to diisonitrile chalkophore deficiency and further indicate that the mycobacterial chalkophore system can utilize nonnative diisonitriles as functional chalkophores.

### PPE1 is required for growth in low copper.

**FIG 6:**
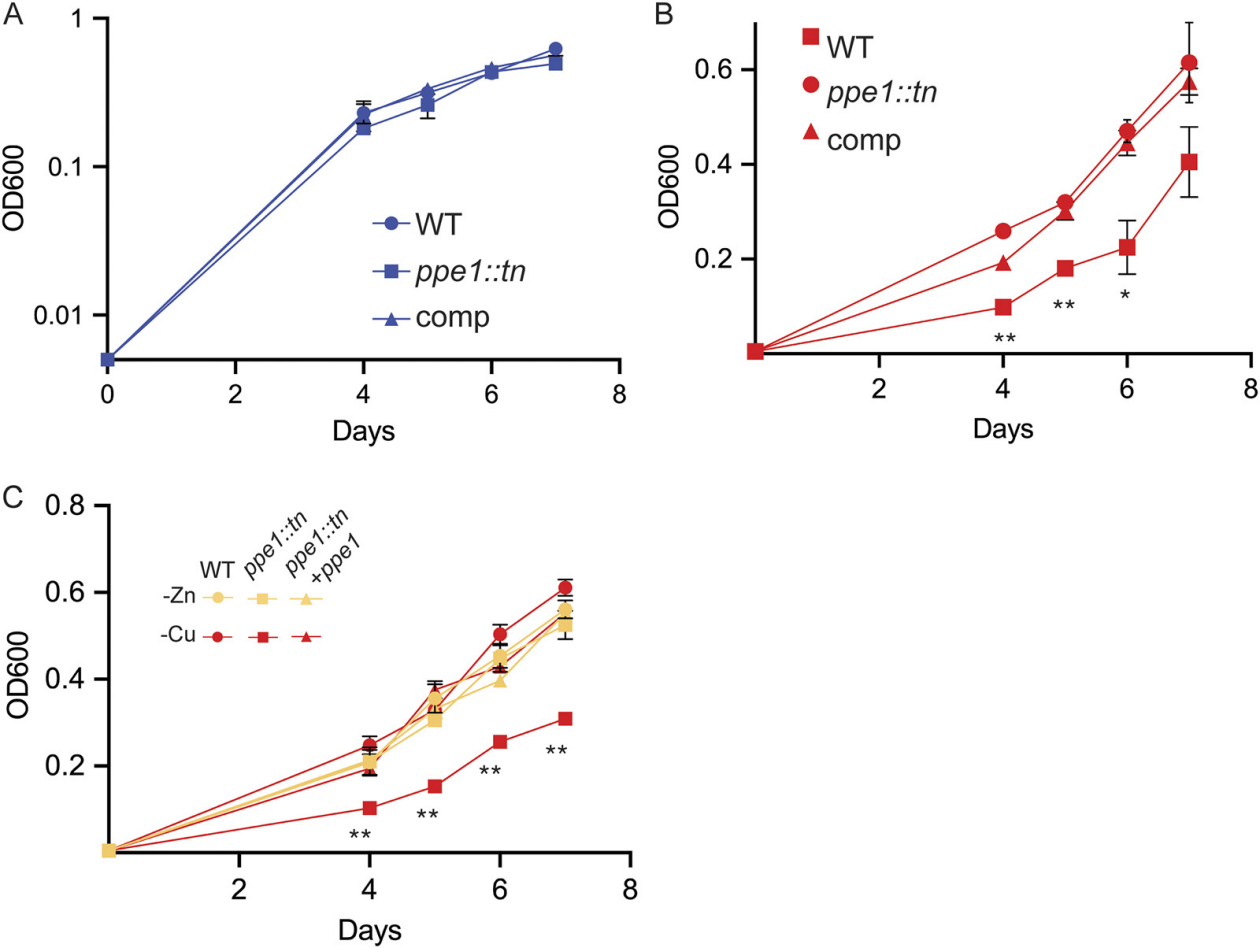
PPE1 is required for chalkophore function. (A) Growth of wild-type, M. marinum *ppe1*::*tn* and *ppe1*::*tn* plus *ppe1* in metal-replete media. (B) Growth of the same strains as in panel A with 10 μM TTM. (C) Growth of wild-type M. marinum (circles), M. marinum Δ*ppe1* (squares) and complemented strain in Zn-deficient (yellow) or Cu-deficient (red) medium. Error bars indicate SEM for all panels.

The first gene in *nrp* gene cluster is *ppe1* (*rv0096c* [Fig. 1A]). The data above indicate that the PPE1 protein and the small RNA *ppe1-5′*, which is at the 5′ end of the *ppe1* locus (19), are both strongly induced by copper starvation (Fig. 2). PPE1 is predicted to be a multipass transmembrane protein, but prior heterologous-expression studies that identified the diisonitrile products of the M. marinum *nrp* locus did not include PPE1, suggesting that it is not required for diisonitrile biosynthesis (25). To determine whether PPE1 is also required for resistance to Cu starvation, we tested M. marinum *ppe1*::*tn* under low-Cu and low-Zn conditions. M. marinum lacking *ppe1* grew identically to wild-type M. marinum in replete medium (Fig. 6A). However, chelation of Cu with TTM impaired growth of M. marinum lacking *ppe1*, a phenotype that was rescued by reintroduction of *ppe1* (Fig. 6B). PPE1 is also required for M. marinum growth under Cu-limiting conditions but not Zn-limiting conditions, a phenotype that resembles loss of *fadD10* or *nrp* (*rv0101*) (Fig. 6C). Loss of *ppe1* severely sensitized M. tuberculosis to both BCS and TTM on agar medium (Fig. S5A). Combined with data above showing that *ppe1* is coregulated by low Cu with the other genes in the chalkophore cluster, these functional data indicate that PPE1 is an integral component of the chalkophore system required for resistance to Cu starvation.

## DISCUSSION

### *nrp*, *fadD10*, and *ppe1* are required for copper acquisition.

Our data strongly support a model in which M. tuberculosis and M. marinum use diisonitrile lipopeptides as a copper acquisition system under low-Cu conditions. Loss of chalkophore biosynthesis through genetic deletion of the *nrp* (*rv0101*) gene, *fadD10*, or *ppe1* confers sensitivity to Cu chelation or Cu starvation. These phenotypes are rescued by a synthetic diisonitrile chalkophore, indicating that the phenotypes of *nrp* cluster mutants are due to deficiency of the diisonitrile product. PPE1 is predicted to be a membrane protein, and we speculate that its role may be in chalkophore transport, as other members of this gene family have been implicated in transport of other nutrients in mycobacteria, including glycerol, phosphate, and heme (37–39). The role of chalkophores in low-Cu growth is thematically similar to the iron acquisition role of siderophores, which are widely utilized by bacterial pathogens to acquire iron within the host. Multiple groups have reported that M. tuberculosis lacking *nrp* is attenuated in mouse models of infection (19, 23), strongly suggesting that M. tuberculosis experiences Cu-limiting environments in the host and that diisonitrile chalkophores serve to counter this host-imposed Cu limitation.

A recent study by Mehdiratta et al. examined the function of the NRPS encoded by *nrp* (*rv0101*) and its natural product in M. tuberculosis (24). The investigators identified the diisonitrile product produced by M. tuberculosis and demonstrated the accumulation of this lipopeptide in infected mouse lung, with a time course of accumulation that matches the pattern of attenuation of M. tuberculosis lacking *nrp*. Importantly, this study identified the previously unknown structure of the M. tuberculosis diisonitrile, which differs from the M. marinum diisonitrile due to differences between the biosynthetic modules of the M. tuberculosis NRPS protein and the M. marinum NRPS. However, our findings and conclusions about the role of diisonitriles in metal acquisition differ substantially from those of Mehdiratta et al., who concluded that the major function of the product of the *nrp* cluster is both to acquire Zn and to resist Zn toxicity. In contrast, our data do not indicate a role of the *nrp* gene cluster in Zn acquisition but indicate an important role in Cu acquisition. There are several methodologic differences that could contribute to our different conclusions. In the work of Mehdiratta et al., the metal limitation assays were performed in Chelex-treated Sauton’s medium, removing all metals, including those ordinarily present in this medium (iron and magnesium), with single metals then added back and bacterial growth measured. Under these conditions, only Zn was able to rescue the defective growth of M. tuberculosis lacking *nrp*; Cu did not. In contrast, our assays use a dropout approach in which all metals are depleted and then replaced, with the exception of individual metals. Thus, M. tuberculosis requires *nrp* to grow when Cu is lacking but can grow when Zn is the only metal provided.

Our data support a primary role for chalkophores in tolerance to copper limitation, rather than zinc. Prior data obtained using synthetic diisonitrile SF2768, and the same compound purified from heterologous expression, indicated that diisonitriles bind Cu, but not Zn, with high affinity. In addition, in *Streptomyces*, SF2768 has been shown to mediate Cu uptake into cells directly (26). Our data support a similar role in M. tuberculosis and M. marinum based on the following observations:

1. The genes in the *nrp* gene cluster are responsive to Cu limitation but not Zn limitation.
2. M. tuberculosis and M. marinum lacking either *nrp*, *fadD10*, or *ppe1* are growth inhibited in Cu- but not Zn-deficient medium or in the presence of Cu but not Zn chelators.
3. The phenotypes of chalkophore-deficient M. tuberculosis and M. marinum are rescued by a synthetic diisonitrile, which binds Cu but not Zn. Although this synthetic diisonitrile differs in structure from the recently identified diisonitrile natural product from M. tuberculosis and the predicted diisonitrile natural product from M. marinum (24, 25), we show that it is utilized as a functional chalkophore by mycobacteria and its defined metal binding specificity for Cu further supports a model in which diisonitriles function as chalkophores.

It remains possible that the structure of the M. tuberculosis diisonitrile product confers some binding to Zn and an ability to mediate its uptake when Zn is the only metal available, a hypothesis that will require total synthesis of the M. tuberculosis natural product. Our data do indicate that expression of PPE1, although primarily responsive to low Cu, is enhanced by low Zn when Cu is also absent, suggesting some interaction between Zn and Cu starvation. Prior data (29), which we reproduce here, indicate that low zinc stimulates expression of *ctpV*, which encodes a copper exporter under the control of CsoR (40–42). This low-zinc response would result in copper efflux, potentially lowering the cytoplasmic copper concentration and explaining an interaction between deprivation of zinc and deprivation of copper.

### Roles of copper acquisition by chalkophores.

The best-studied chalkophore systems are encoded by *Methanococcus*. The chalkophores in methanococci are ribosomally produced, posttranslationally modified peptides that bind copper with high affinity (43, 44). *Methanococcus* chalkophores mediate Cu import and eventual loading onto the membrane-bound Cu-dependent enzyme particulate methane oxygenase. Although the mycobacterial chalkophores differ in chemical structure and Cu binding chemistry, the mycobacterial diisonitriles appear to play similar roles in Cu acquisition. However, the critical cellular function of Cu that is maintained by the mycobacterial chalkophore system under conditions of Cu limitation, both *in vitro* and in the host, remains to be determined. Given the requirement for the chalkophore system in M. tuberculosis virulence, the findings reported here may suggest that Cu limitation is imposed by the host during infection. This conclusion adds complexity to the existing paradigm that high-Cu stress imposed on phagosomal pathogens in macrophages is the most relevant Cu component of nutritional immunity. Direct measurements of the metal concentrations in M. tuberculosis containing phagosomes of murine macrophages have revealed Cu concentrations that range from approximately 400 μM immediately after infection to 25 μM after 24 h (45), concentrations that are sufficient to induce the Cu-responsive CsoR regulon, indicating that M. tuberculosis experiences high Cu in macrophages (18). Zn concentrations are approximately 40 μM immediately after infection, rising to ~426 μM 24 h after infection (45), a concentration that is sufficient to induce the Zn efflux pump CtpC (18). The role of the *nrp*-dependent diisonitrile natural products in Cu acquisition, but not resistance to Cu toxicity, may suggest that Cu starvation paradoxically coexists with high Cu stress during infection. Additional support for the importance of the Cu starvation response in M. tuberculosis pathogenesis comes from analysis of M. tuberculosis lacking the SigC alternative sigma factor (46). M. tuberculosis lacking *sigC* is sensitive to Cu, but not Zn, limitation, underexpresses the chalkophore cluster genes in low Cu, and is attenuated in SCID mice (46). Low and high Cu stresses could be imposed on M. tuberculosis in distinct cellular or anatomic compartments, imposed by distinct effectors of host immunity, or in distinct phases of infection (i.e., acute versus chronic versus latent). Tissue-specific differences in Cu availability have been found in Candida albicans infection, in which infection of the kidney imposes a Cu limitation response and infection of the blood imposes a high Cu response (47). Similarly, although Zn and Cu intoxication is imposed on M. tuberculosis in macrophages (18, 20, 21), the caseum in which M. tuberculosis resides in nonhuman primate lung cavities is rich in calprotectin (29), which can bind multiple metals (48, 49), supporting the idea the metal excess and metal limitation may coexist in M. tuberculosis infection.

In summary, the data presented here show that diisonitrile lipopeptides produced by the *nrp* locus function as chalkophores in copper acquisition and will empower further investigation of the roles of chalkophores and Cu restriction in nutritional immunity to M. tuberculosis.

## MATERIALS AND METHODS

### Reagents.

Middlebrook 7H10 agar, 7H9 broth, dextrose, Tween 80, bovine serum albumin (BSA), and UltraPure DNase/RNase-free distilled water were purchased from Fisher Scientific. Ammonium tetrathiomolybdate (TTM), *N*,*N*,*N*′,*N*′-tetrakis(2-pyridylmethyl)ethylenediamine (TPEN), dimethyl sulfoxide (DMSO), copper sulfate, zinc sulfate, magnesium sulfate, and calcium chloride were purchased from Millipore Sigma. Biotechnology (BT) grade Chelex 100 resin, sodium form, was purchased from Bio-Rad. The synthetic diisonitrile chalkophore was synthesized and characterized as previously described (compound 4 in reference 27).

### General growth conditions, strains, and DNA manipulations.

M. tuberculosis Erdman and M. marinum M strain were grown and maintained in 7H9 medium (broth) or on 7H10 agar supplemented with 10% oleic acid-albumin-dextrose-catalase (OADC) and 0.05% glycerol (7H9 OADC/7H10 OADC). Broth cultures were additional supplemented with 0.02% Tween 80. Genetic mutations were generated by specialized transduction utilizing the temperature-sensitive phage phAE87 (50). Mutant strains were confirmed by PCR (see Fig. S2 in the supplemental material) followed by sequencing. M. marinum transposon mutants were a generous gift from the Tobin Lab at Duke University. For a complete strain list with relevant features, see Table S1. Plasmids utilized in this study were generated using standard molecular techniques and are listed with their features in Table S2.

10.1128/mbio.02513-22.6 TABLE S1 Bacterial strains used in this work. Download Table S1, XLSX file, 0.01 MB. Copyright © 2022 Buglino et al. 2022 Buglino et al. https://creativecommons.org/licenses/by/4.0/ This content is distributed under the terms of the Creative Commons Attribution 4.0 International license.

10.1128/mbio.02513-22.7 TABLE S2 Plasmids used in this work. Download Table S2, XLSX file, 0.01 MB. Copyright © 2022 Buglino et al. 2022 Buglino et al. https://creativecommons.org/licenses/by/4.0/ This content is distributed under the terms of the Creative Commons Attribution 4.0 International license.

### Chelated copper- and zinc-depleted 7H9 media and buffers.

Ion-deficient buffers were generated by treatment with 10 g/L of biotechnology-grade Chelex resin for 24 h at room temperature, followed by 0.2-μm filtration.

7H9 medium was generated from primary components as follows: 0.5 g/L of ammonium sulfate, 1 g/L of monopotassium phosphate, 2.5 g/L of disodium phosphate, 0.1 g/L of sodium citrate, 0.5 g/L l-Glutamic acid, 0.001 g/L of pyridoxine hydrochloride., and 0.0005 g/L of biotin were dissolved in 850 mL of deionized water. Once dissolved, 10 mL of 50% glycerol, 2.5 mL of 20% Tween 80, and 100 mL of ADS (albumin, dextrose, and saline) supplement were added and the total volume was adjusted to 990 mL with deionized water. The resulting solution was then treated with 10 g/L of Chelex resin for 24 h at 4°C. Resin was removed by 0.2-μm filtration, and iron, magnesium, and calcium levels were restored to 0.04 g/L, 0.05 g/L, and 0.0005 g/L, respectively, by the addition of 4, 5, and 0.05 mL of 10-mg/mL sterile filtered solutions of ferric ammonium citrate, magnesium sulfate, and calcium chloride dissolved in UltraPure distilled water. Sterile filtered 10-mg/mL solutions of copper sulfate and/or zinc sulfate dissolved in UltraPure distilled water were used to restore Cu and/or Zn levels to 0.001 g/L when indicated.

### Cation limitation and chelation sensitivity growth assays.

For liquid growth assays, the indicated strains were pregrown in nonchelated medium until reaching an optical density at 600 nm (OD_600_) of ~1.0. Cells were then collected by centrifugation (3,700 × *g*, 10 min) and washed twice with chelated phosphate-buffered saline plus 0.02% Tween 80 (PBS-Tween 80). Growth assays were initiated at a calculated OD_600_ of 0.005 by the addition of 1 mL of washed culture at an OD_600_ of 0.05 to 9 mL of chelated 7H9-ADS deficient in either copper or zinc ions or in chelated 7H9-ADS replete for all ions. Selective ion chelators and synthetic chalkophore analogues were assayed in chelated 7H9-ADS replete for all ions. M. tuberculosis and M. marinum growth assays were conducted at 37°C and 30°C, respectively, with growth assayed via daily OD_600_ measurements. To maintain logarithmic growth and ensure that all cells entered log phase to remove any confounding effects of extended lag phase of any mutant or condition, cultures were diluted 1:10 upon reaching an OD_600_ of 0.4, and thus, the *y* axes of many growth curve plots show “dilution corrected OD_600_” that incorporates this dilution factor.

For M. marinum and M. tuberculosis TTM survival assays, cells were pregrown and washed as indicated above. Replicate 2-mL tubes containing chelated 7H9-ADS replete for all ions and the indicated concentrations of TTM, or a DMSO vehicle control, were inoculated at an OD_600_ of 0.01. After 7 days of incubation at 30°C (M. marinum) or 37°C (M. tuberculosis), aliquots were removed and plated on nonchelated 7H10-OADC plates to determine growth/survival. CFU per microliter of culture were determined after 7 days of incubation at 30°C (M. marinum) or 21 days at 37°C (M. tuberculosis).

### RNA extraction and RT-qPCR.

For TTM-stimulated RT-qPCR, the indicated strains were pregrown and washed as described above. Triplicate 30 -mL cultures of chelated 7H9-ADS replete for all ions were then inoculated at an OD_600_ of 0.02. Upon reaching an OD_600_ of 0.6 to 0.7 cultures were treated with the indicated concentration of TTM, or a DMSO vehicle control, for 5 h. Cells were then collected by centrifugation (3,700 × *g*, 10 min), suspended, and stored in 1 mL of TRIzol reagent for downstream RNA extraction and processing described below.

For ion-deficient RT-qPCR assays, 100-mL cultures of M. tuberculosis (Erdman) were pregrown in nonchelated 7H9 OADC medium to an OD_600_ of ~2.0. Cells were collected by centrifugation (3,700 × *g*, 10 min), washed once in chelated PBS-Tween 80, and concentrated to an OD_600_ of 6.0. Triplicate cultures were then inoculated at a starting OD_600_ of 0.2 by the addition of 1 mL of washed culture to 29 mL of chelated 7H9-ADS containing the indicated complement of ions. At 48 h after inoculation, cells were collected by centrifugation (3,700 × *g*, 10 min), suspended, and stored in 1 mL of TRIzol reagent for downstream RNA extraction and processing described below.

Cells in 1 mL of TRIzol were mechanically disrupted with zirconia bead via three 30-s pulses in a BioSpec Mini24 BeadBeater. Following lysis, beads were removed by centrifugation at 20,000 × *g* for 5 min and total RNA was isolated using the Direct-zol RNA miniprep kit (Zymo Research) as directed by the manufacturer. Contaminating genomic DNA was removed using the Turbo DNA-free kit (Invitrogen). A total of 500 ng of resulting total RNA was used to synthesize cDNA via random priming utilizing the Maxima H-Minus cDNA synthesis kit (Thermo Fisher). Real-time qPCR was performed on a 7500 real-time PCR system (Applied Biosystems). The amplification product was detected by SYBR green using the Dynamo Flash qPCR kit (Thermo Fisher). For each gene of interest (GOI), normalized cycle threshold (*C_T_*) was determined relative to the housekeeping gene *sigA*. Relative expression level was calculated using the formula 2^−(CTGOI-CT^*^sigA^*^)^. Primer sets used to amplify individual GOI are listed in Table S3.

10.1128/mbio.02513-22.8 TABLE S3 Oligonucleotides used in this work. Download Table S3, XLSX file, 0.01 MB. Copyright © 2022 Buglino et al. 2022 Buglino et al. https://creativecommons.org/licenses/by/4.0/ This content is distributed under the terms of the Creative Commons Attribution 4.0 International license.

### Immunoblotting.

Lysates for immunoblotting were prepared from duplicate cultures of the indicated strains and washed and treated as described for RNA extraction above, except that postcentrifugation pellets were washed once with 1 mL of lysis buffer (350 mM sodium chloride, 20 mM Tris [pH 8.0], 1 mM 2-mercaptoethanol) prior to suspension in 0.8 mL of lysis buffer plus ~100 μL of zirconia beads. Lysis was performed by three 45-s pulses in a BioSpec Mini24 BeadBeater with 5-min intervening rest periods on ice. Beads and debris were removed by centrifugation at 20,000 × *g* for 15 min at 4°C, and the resulting supernatant was mixed 1:1 with 2× Laemmli sample buffer supplemented with 0.1 M dithiothreitol (DTT). Twenty microliters of each sample, heated for 10 min at 100°C, was then separated on 4 to 12% NuPAGE bis-Tris polyacrylamide gels. Separated proteins were transferred to nitrocellulose and probed with the appropriate antibodies. Antibodies used in this study were monoclonal anti-E. coli RNA polymerase β (BioLegend), rabbit polyclonal anti-GFP (Rockland), and sdAb anti-ALFA tag-horseradish peroxidase (HRP) (NanoTag Biotechnologies). PPE1-GFP blots were quantitated using ImageJ software. Relative GFP signal was normalized to corresponding Rpoβ loading control levels and DMSO or replete-ion complement controls.

### Data availability.

All strains used in this work are available from the corresponding author upon request. There is no external data set associated with the paper. The uncropped blots for Fig. 2B and C and Fig. 4E are available for review upon request and were included in the submission materials for the manuscript.
