# Supplementary figures and images for: Diisonitrile Lipopeptides Mediate Resistance to Copper Starvation in Pathogenic Mycobacteria
Source: mBio. 2022 Oct 5;13(5):e02513-22. doi: 10.1128/mbio.02513-22 (PMC9600254; doi:10.1128/mbio.02513-22)

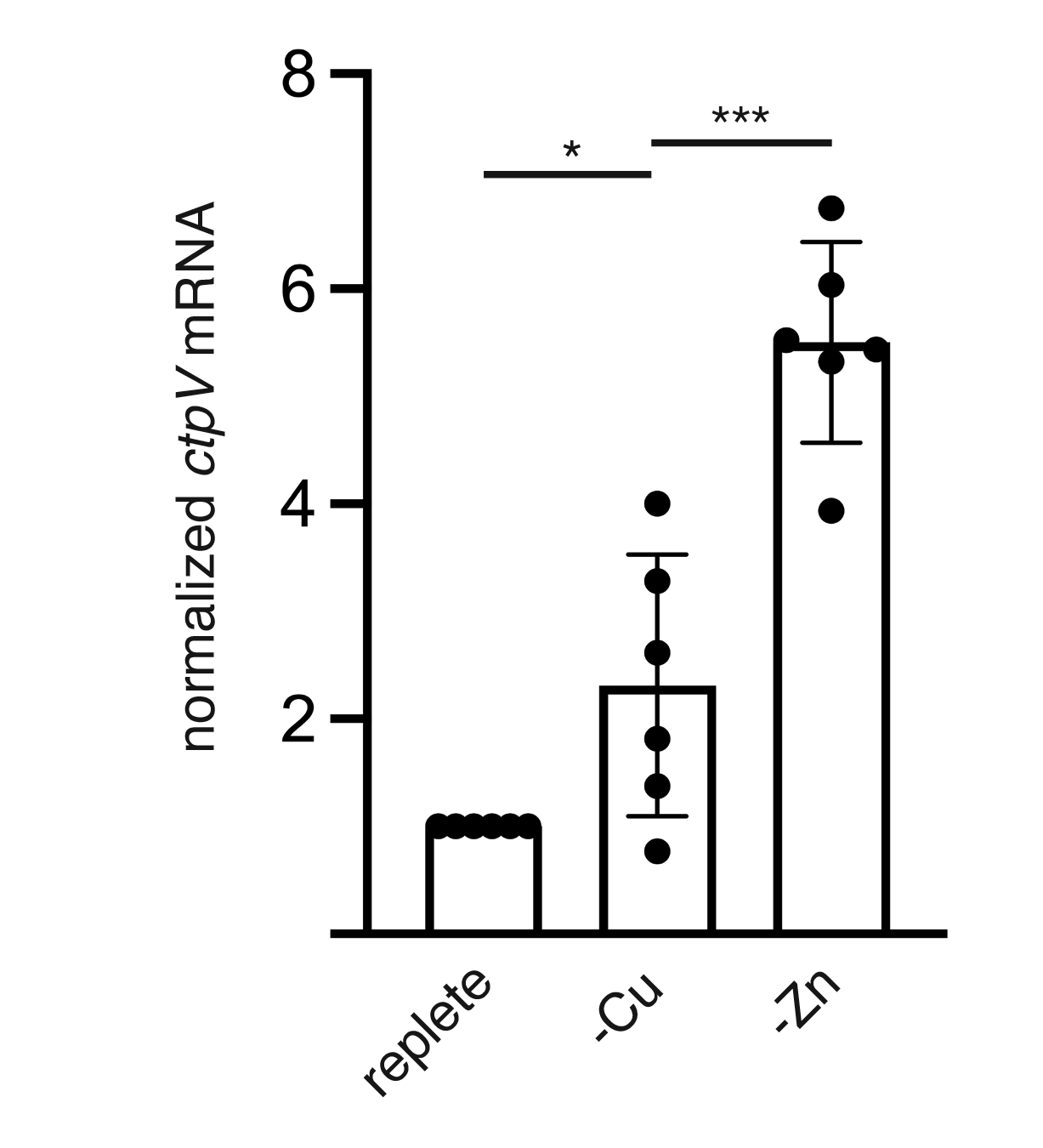

Supplement: FIG S1 [file mbio.02513-22-s0001.tif]

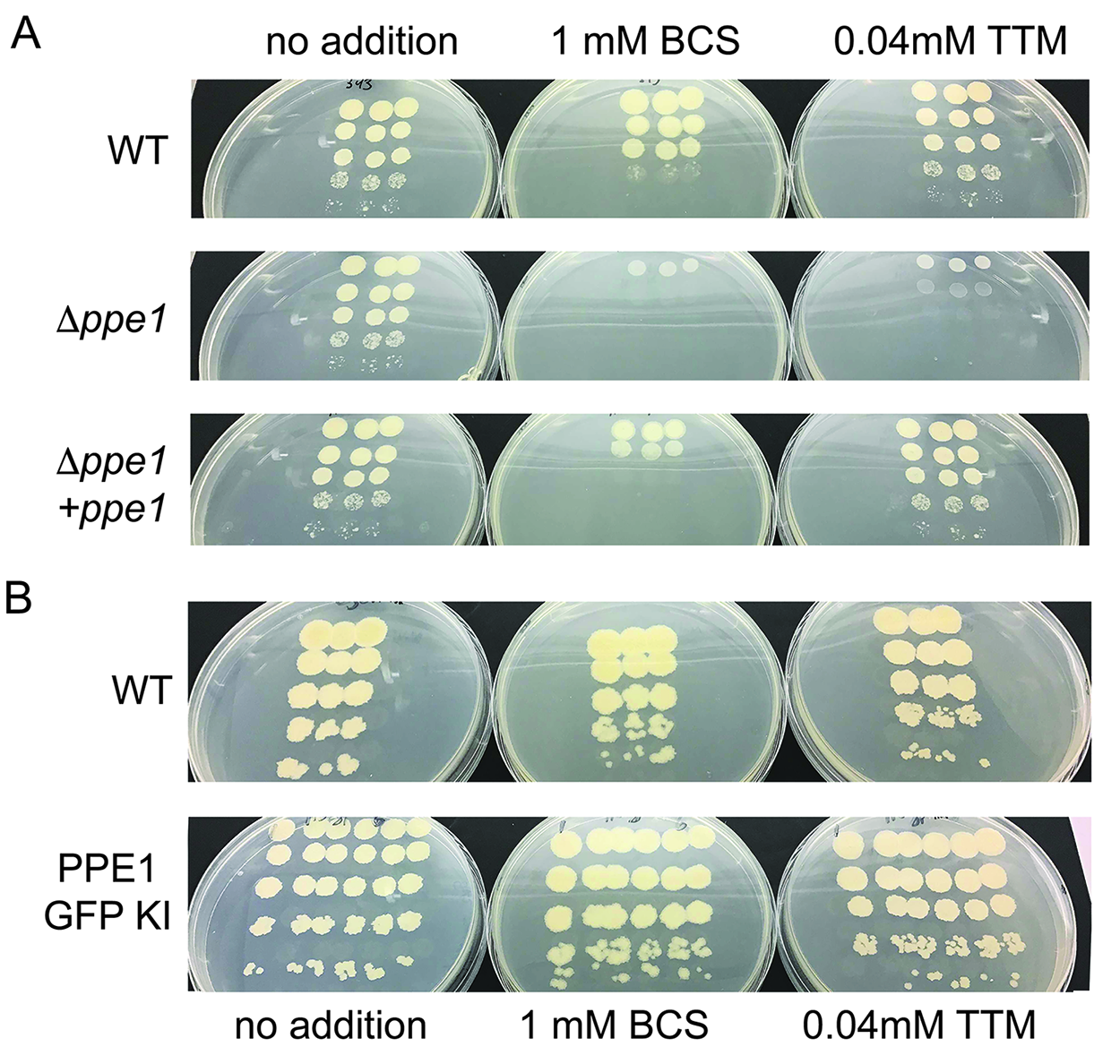

Supplement: FIG S5 [file mbio.02513-22-s0005.tif]

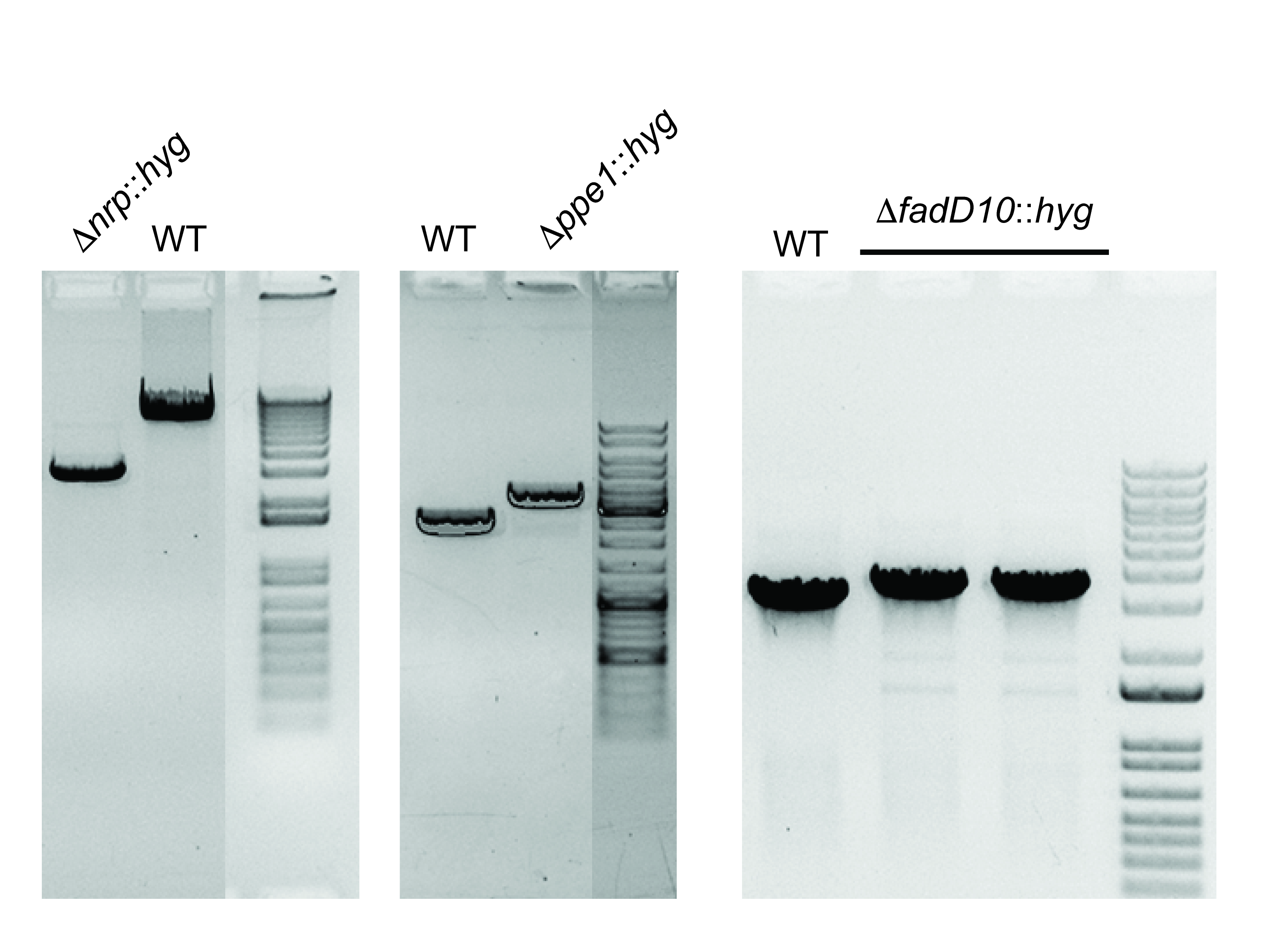

Supplement: FIG S2 [file mbio.02513-22-s0002.tif]

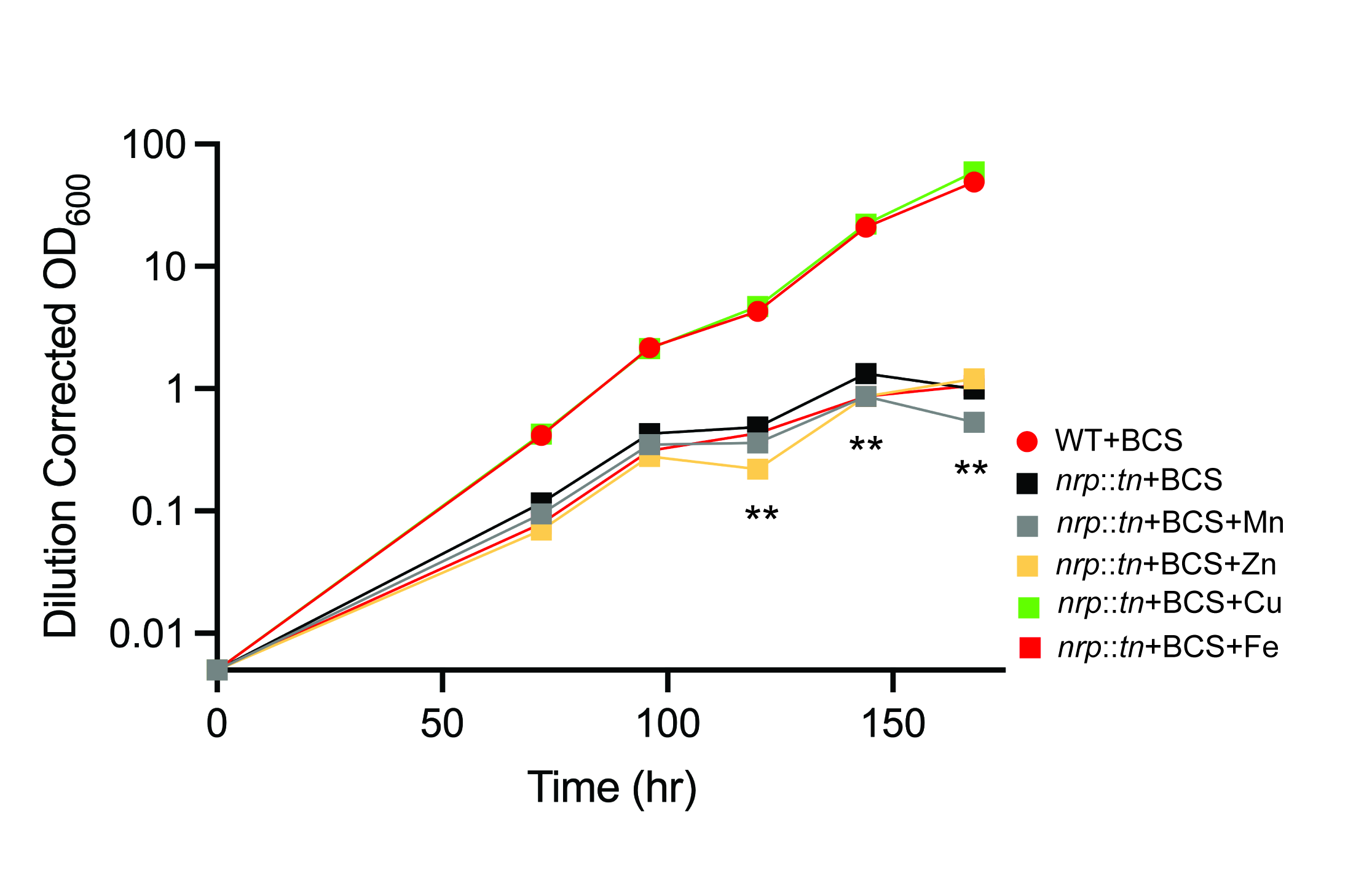

Supplement: FIG S3 [file mbio.02513-22-s0003.tif]

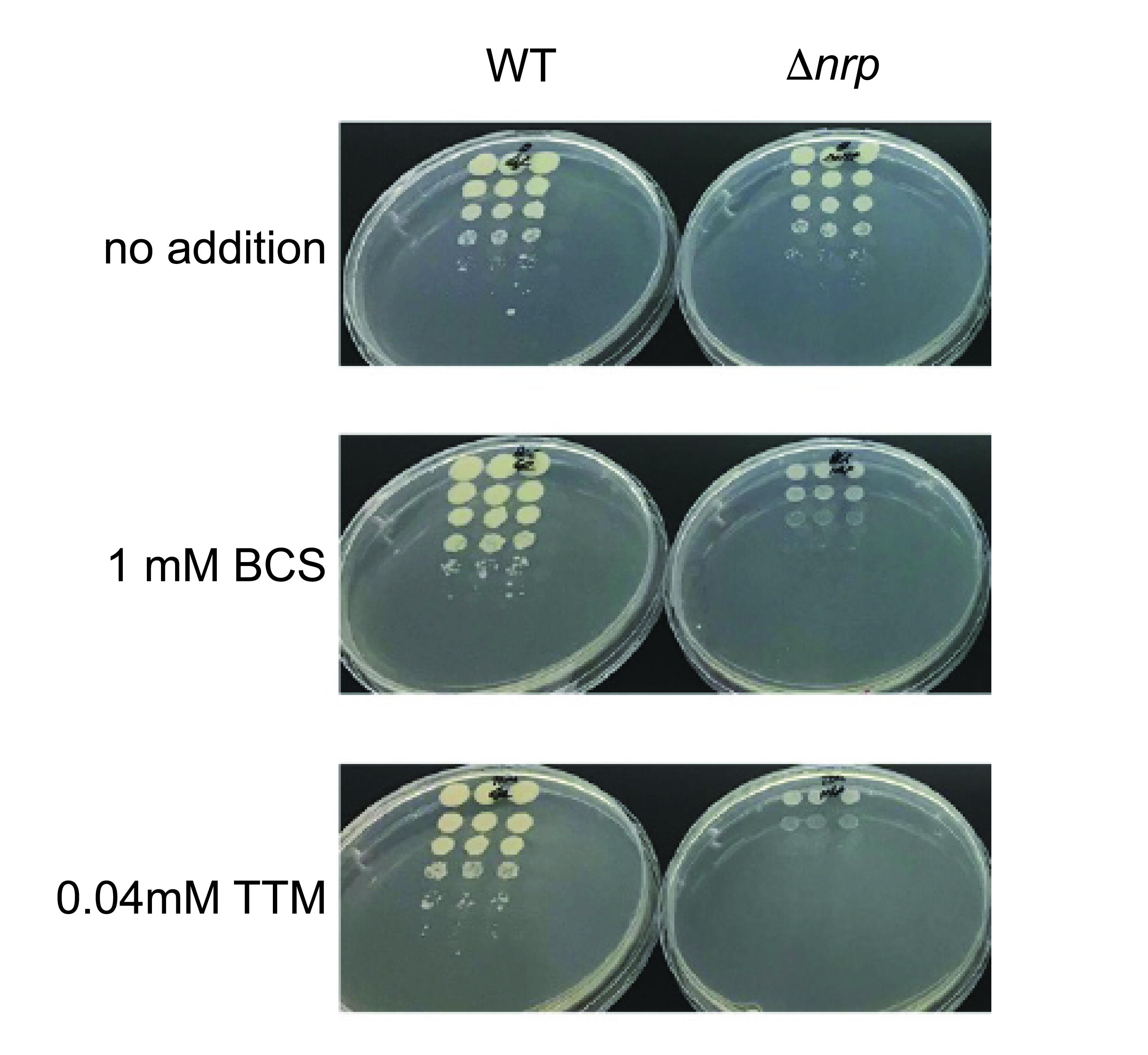

Supplement: FIG S4 [file mbio.02513-22-s0004.tif]
